# Increased expression of class III *β*-tubulin in castration-resistant human prostate cancer

**DOI:** 10.1038/sj.bjc.6605245

**Published:** 2009-08-18

**Authors:** S Terry, G Ploussard, Y Allory, N Nicolaiew, F Boissière-Michot, P Maillé, L Kheuang, E Coppolani, A Ali, F Bibeau, S Culine, R Buttyan, A de la Taille, F Vacherot

**Affiliations:** 1 INSERM, Unité 955, Créteil F-94000, France; 2 Université Paris 12, Faculté de Médecine, Créteil F-94000, France; 3 Université Paris-Sud 11, Faculté de Médecine, Le Kremlin-Bicêtre F-94276, France; 4 AP-HP, Groupe Henri Mondor-Albert Chenevier, Service de Pathologie et Urologie, Créteil F-94000, France; 5 Centre Val d’Aurelle, Service de Pathologie, Montpellier F-34298, France; 6 Cancer Center, The Ordway Research Institute, Albany, NY 12208, USA

**Keywords:** class III *β*-tubulin, prostate cancer, hormone therapy, disease progression, therapeutic resistance

## Abstract

**Background:**

Class III *β*-tubulin (*β*III-tubulin) is expressed in tissues of neuronal lineage and also in several human malignancies, including non-small-cell lung carcinoma, breast and ovarian cancer. Overexpression of *β*III-tubulin in these tumours is associated with an unfavourable outcome and resistance to taxane-based therapies. At present, *β*III-tubulin expression remains largely uncharacterised in prostate cancer.

**Methods:**

In this report, we evaluated the expression of *β*III-tubulin in 138 different human prostate tumour specimens by immunohistochemistry from patients with hormone-treated or hormone-untreated prostate cancer. *β*III-tubulin expression was also examined in various prostatic cancer cell lines including in androgen-sensitive human prostate cancer cells, LNCaP, grown in androgen-depleted medium in 2D cultures or as tumour xenografts when the host mouse was castrated.

**Results:**

Whereas moderate-to-strong *β*III-tubulin expression was detected in only 3 out of 74 (4%) hormone-naive tumour specimens obtained from patients who never received hormone therapy, 6 out of 24 tumour specimens (25%) from patients treated for 3 months with neoadjuvant hormone therapy and 24 out of 40 (60%) castration-resistant tumour specimens from chronic hormone-treated patients were found to express significant levels of *β*III-tubulin. These findings were supported by *in vitro* and *in vivo* settings.

**Conclusion:**

Our data indicate that *β*III-tubulin expression is augmented in prostate cancer by androgen ablation and that the expression of this *β*-tubulin isoform is associated with the progression of prostate cancer to the castration-resistant state, a stage largely responsible for mortality from prostate cancer.

Prostate carcinoma (PCa) is the most common malignancy and the second leading cause of cancer death in men (Ferlay *et al*, 2007; Jemal *et al*, 2008). Despite the current widespread use of prostate-specific antigen (PSA) screening that increases the detection of PCa at an early stage when it is localised to the prostate, ∼30% of men treated with surgical or radiation therapies will relapse (Han *et al*, 2001). These men and those who present with locally advanced or overt metastatic PCa are treated by hormonal therapies that deplete circulating androgen levels (Hellerstedt and Pienta, 2002). For most men with advanced PCa, hormone therapy provides a median progression-free survival of 12–33 months until the emergence of a castration-resistant prostate cancer (CRPC) that is refractory to the low androgen levels of the hormone-treated patient. As CRPC generally responds poorly to alternate therapeutics, this form of the disease is overwhelmingly responsible for mortality from PCa.

Recently, however, treatments involving docetaxel-based chemotherapy have shown some benefit for CRPC, although the survival advantage remains relatively limited (Petrylak *et al*, 2004; Tannock *et al*, 2004). Taxanes, of which docetaxel is a derivative, target the microtubules of cancer cells to disrupt the mitotic apparatus leading to cancer cell death. Microtubules are composed of polymers of *α*- and *β*-tubulin heterodimers (Orr *et al*, 2003; Seve and Dumontet, 2008). Both *α*- and *β*-tubulins exist as multiple isotypes with a complex pattern of distribution among different tissues. Class III *β*-tubulin (*β*III-tubulin) is normally expressed in neuronal tissues and in neuroendocrine (NE) cells, and this isoform of *β*-tubulin is also expressed in some solid tumours, including tumours of neuronal origin and non-small-cell lung, gastric, breast and ovarian carcinomas. For these latter tumours, the expression of *β*III-tubulin correlates with a poor overall survival as well as with reduced response to taxanes (Ferrandina *et al*, 2006; Gan *et al*, 2007; Galmarini *et al*, 2008; Seve and Dumontet, 2008). Indeed, resistance to taxanes can be induced in cultured human tumour cells by transfection with *β*III-tubulin, whereas *β*III-tubulin depletion in cells resulted in the sensitisation to taxanes (Lu and Luduena, 1993; Panda *et al*, 1994; Kavallaris *et al*, 1999; Kamath *et al*, 2005; Shalli *et al*, 2005; Gan *et al*, 2007). These findings suggest that the presence of *β*III-tubulin in cancer cell microtubules alters their sensitivity to taxane-based agents.

At present, the expression of *β*III-tubulin remains relatively uncharacterised in PCa. One study did assess *β*III-tubulin expression in a small group of localised PCa specimens by immunohistochemistry and reported that *β*III-tubulin was moderately expressed in both benign and malignant tissues cells of the prostate (Ranganathan *et al*, 1997). However, this evaluation did not include analysis of castration-resistant disease, which is the actual candidate for standard chemotherapeutic intervention. In this study, we provide evidence, on the basis of immunostaining profiles of different classes of prostate tumours, that *β*III tubulin expression is highly upregulated in prostate cancer cells during the transition from hormone-sensitive to the castration-resistant state. Whereas this characterisation might help to better explain the more aggressive and therapeutic-resistant nature of CRPC, it also has implications for the use of alternative microtubule-targeting therapeutic agents that might be more effective than docetaxel in the treatment of CRPC.

## Materials and methods

### Human prostate tissue samples

Formalin-fixed, paraffin-embedded prostate tissues were obtained from the Department of Pathology at the Henri Mondor Hospital and from the Cancer Centre in Montpellier, France. Pathological specimens of hormone-naive (HNPC; *n*=74) and hormone-therapy-treated (HTPC; *n*=24) prostate cancers used in this study were obtained from patients treated for localised disease by radical prostatectomy. Hormone-therapy-treated prostate cancer cases comprised patients who had been treated with 3 months of neoadjuvant therapy with LHRH agonists before surgery. Metastatic CRPC (*n*=6) tissues were liver biopsy samples collected from patients diagnosed with liver metastases as the first site of metastatic disease. Primary CRPC specimens (*n*=34) were collected at the time of the trans-urethral resection of the prostate in patients who experienced disease recurrence after an initial response to hormone therapy. Recurrence was defined as patients having PSA progression despite a complete androgen blockade therapy and a castrated level of testosterone. All samples were obtained with institutional review board approval from the respective institutions.

### Immunohistochemical analysis

Immunohistochemistry was carried out on tissue microarrays (TMAs) containing samples (primary CRPC and HNPC cases) and regular sections (HNPC (10 cases), primary CRPC, HTPC and biopsy samples). For TMA, four replicate cores (with diameter of 0.6 mm) were collected from the donor block. *β*III-tubulin protein expression was assessed after standard biotin–avidin complex immunohistochemistry using a monoclonal antibody against *β*III-tubulin (1 : 500; clone TUJ1; Covance, Emeryville, CA, USA). Protein expression was scored as null (0), weak (1), moderate (2) and strong (3). In this analysis, a case was considered positive only when the score was ⩾2 in more than 10% of cancer cells, whereas cases with <10% staining or those that scored <2 were considered negative. *β*III-tubulin expression was evaluated separately by one pathologist (YA) and by two non-pathologists (ST and GP). The staining assessment was highly reproducible. Immunostains obtained from regular sections of CRPC and HNPC samples were constantly comparable with those obtained in the TMA analysis. Positivity in nerves and axons present in the sections served as positive control. Negative controls were included in each experiment in which the primary antibody was omitted. The absence of immunostaining in red cells was also used as a negative control as previously described (Ranganathan *et al*, 1997). The following antibodies were used to assess the NE characteristics of the specimens: polyclonal antibodies to chromograninA (CgA; Dako, Trappes, France; A0430); monoclonal antibodies to synaptophysin (Snp88, Biogenex, San Ramon, CA, USA) and NSE (BBS/NC/VI-H14, Dako).

### Cell culture

Human prostate cancer cell lines LNCaP (clone FGC), 22Rv1, PC3 and DU145 were obtained from American Type Culture Collection (Manassas, VA, USA), and the cells were maintained in RPMI 1640 supplemented with 10% fetal bovine serum (FBS) and penicillin/streptomycin. For androgen-reduced conditions, LNCaP cells were cultured in phenol red-free RPMI supplemented with 10% charcoal-stripped FBS for the indicated time in the presence or absence of Dihydrotestosterone (DHT, Sigma-Aldrich, Saint-Quetin Fallavier, France).

### Western blot analysis

Protein lysates were prepared in the RIPA buffer (radioimmunoprecipitation assay lysis buffer) supplemented with protease inhibitor cocktail (Roche Diagnostics, Basel, Switzerland) and phosphatase inhibitors (25 mmol l^−1^ orthovanadate and 50 mmol l^−1^ NaF) (Sigma-Aldrich). The total protein concentration of the soluble extract was determined using the Bicinchoninic Acid Kit (Sigma-Aldrich). Each protein sample (30 *μ*g) was resolved to SDS–PAGE, transferred onto a polyvinylidene difluoride membrane (Millipore, Molsheim, France) and incubated with a monoclonal antibody against *β*III-tubulin (1 : 10 000) or *β*-actin (1 : 16 000; AC-15; Sigma-Aldrich). Immune complexes were visualised by enhanced chemiluminescence detection (ECL plus kit, GE Healthcare, Little Chalfont, UK).

### cDNA synthesis and real-time PCR

Quantitative PCR was carried out using SYBR Green dye on an Applied Biosystems 7000 Real Time PCR system (Applied Biosystems, Foster City, CA, USA). The conditions for RT–PCR have been described previously (Azoulay *et al*, 2008). The amount of *β*III-tubulin mRNA levels relative to the housekeeping gene Ribosomal Protein, large, P0 (RPLP0) was determined on the basis of the comparative threshold cycle CT method (2^−ΔΔCT^). The primer sequences for *β*III-tubulin and RPLP0 have been described previously (Azoulay *et al*, 2008; Frigo and McDonnell, 2008).

### Tumour xenografts in athymic mice

2 × 10^6^ LNCaP cells at low passage (P29) were inoculated s.c. with 0.1 ml of Matrigel (Becton Dickinson Labware, Le-Pont-de-Claix, France) in the flank region of 6-week-old male athymic nude mice (Elevage Janvier – Le Genest-Saint-Isle, France) under anaesthesia. Mice bearing tumours between 200 and 300 mm^3^ were killed or were surgically castrated through scrotal incision. At the indicated days, LNCaP xenografts were processed after killing to evaluate *β*III-tubulin expression. All animal procedures were carried out according to local guidelines on animal care and with appropriate institutional certification.

### Statistical analysis

χ^2^ or Fisher's exact tests were used to assess associations between hormone and *β*III-tubulin status in human specimens. For *post hoc* comparisons, the Bonferroni correction was applied. To evaluate trend over hormone status, *χ*^2^ for trend was calculated. Immunochemistry scoring results of xenografts LNCaP tumours stained for *β*III-tubulin were analysed using Kruskal–Wallis and Mann–Whitney tests. All statistical tests used a two-tailed *α*=0.05 level of significance and were conducted using SAS statistical software, version 9.1 (SAS Institute Inc., Cary, NC, USA).

## Results

### Expression of *β*III tubulin is predominantly expressed in castration-resistant human prostate cancer

A total of 138 different cases of prostatic carcinoma were evaluated in this study. The tumours included 74 specimens of HNPC found in prostatectomy specimens obtained from patients who had never received any form of hormonal therapy, 24 cases of HTPC specimens from patients who received 3 months of adjuvant hormonal therapy before radical prostatectomy and 40 cases of CRPC specimens of which 34 were surgically removed from the prostate to relieve bladder outlet obstruction and 6 cases of castration-resistant liver metastases. All specimens were present in formalin-fixed and paraffin-embedded surgical tissues. The tissues were sectioned and were subsequently immunostained for *β*III-tubulin using a standard immunoperoxidase-based procedure.

**Figure 1:**
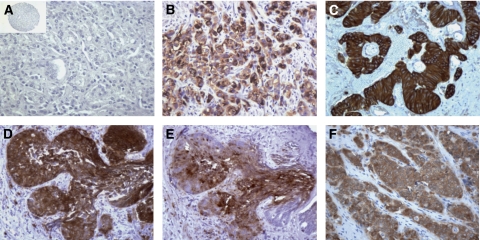
*β*III-tubulin expression in prostate cancers. (**A**–**C**) Representative tissue microarray element, regular section or biopsy sample stained with antibody to *β*III-tubulin with immunostains showing the absence of staining in hormone-naive prostate cancer (panel A) and strong staining in primary (panel B) and metastatic (panel C) castration-resistant refractory prostate cancers. (**D** and **E**) Representative consecutive sections stained with antibodies to *β*III-tubulin (panel D) or NSE (panel E). Immunostainings show concomitant *β*III-tubulin and NSE expression in prostate cancer cells. (**F**) Prostatic small cell carcinoma showing strong immunoreactivity for *β*III-tubulin. Original magnification × 200; inset, × 25.

In the HNPC group, accounting for more than half of the specimens, the presence of *β*III-tubulin expression in cancer cells was relatively rare (Table 1 and Figure 1A). Of these 74 cases, 67 were scored as completely negative (0) and only 3 cases (4%) were considered to be positive, scoring from moderate-to-strong for *β*III-tubulin in the cancer cells. With the exception of occasional stained cells in the basal compartment, *β*III-tubulin expression was not detected in either normal basal cells or luminal prostate epithelial cells present in these specimens. The extremely small number of positively stained specimens in this group prevented us from deriving any statistically reliable association with other patient prognostic factors. Of the 24 cases of HTPC evaluated in our study, 6 (25%) were considered to be positive with *β*III-tubulin immunoreactivity in the moderate-to-strong range in more than 10% of cancer cells (Table 1). Nine cases of HTPC showed complete negative (0) staining, whereas nine other cases in this category were scored as weak or contained only rare (<10%) tumour cells positively stained, and this later group was also recorded as negative. For the overall HTPC group, *β*III-tubulin expression was found to be significantly higher when compared with the HNPC group as evaluated by Fisher's exact test applying the Bonferroni correction (*P*=0.0186).

Finally, of the 40 specimens of CRPC that were analysed, the majority (24 or 60%) was considered to be positive (Figure 1A and C). The predominant staining pattern for CRPC specimens was strong and diffuse. Only three cases of CRPC showed a complete absence of staining (see Supplementary Table 1). Statistical analysis of the data showed that the expression of *β*III-tubulin was significantly correlated with the castration-resistant phenotype (*P*<0.000001). A further analysis with a trend test also reached statistical significance (*P*<0.0001). It is noteworthy that, *β*III-tubulin protein was also expressed in cancer cells of metastatic CRPC lesions present in the liver of patients (Figure 1C). Indeed, the fact that we were able to detect *β*III-tubulin-positive cancer cells in at least five of six cases of this class suggests that the deregulated expression of *β*III-tubulin in CRPC disease is not restricted to recurrent lesions localised to the prostate. This finding is also important because metastatic lesions are mainly responsible for both morbidity and mortality of CRPCs. Cumulatively, these results indicate that *β*III-tubulin expression in human prostate cancer cells is upregulated by hormone therapy and increased further when tumours progress to a castration-resistant stage.

### Expression of *β*III tubulin is upregulated by hormone therapy in human prostate cancer LNCaP cells and LNCaP tumours

*β*III-tubulin is commonly expressed in normal neural tissues and in NE cells (Katsetos *et al*, 2000, 2003). In line with this information, the results showing that *β*III-tubulin expression that was upregulated in PCa cells obtained from both acute and chronic hormone-treated patients might be a manifestation of the NE trans-differentiation phenomenon that has been associated with prostate cancer cells capable of surviving in a low-androgen environment (Yuan *et al*, 2007). For further exploration of this event, 22 *β*III-tubulin-positive CRPC specimens were immunostained with antibodies directed against NE markers; namely CgA, synaptophysin (SYN) and NSE. In 6 out of 22 cases analysed, we were able to detect areas showing strong positivity for both *β*III-tubulin and NSE (Figure 1D and E). In line with this finding, 2 out of 10 evaluable cases demonstrated concomitant *β*III-tubulin and SYN expression. However, we failed to find any co-expression pattern when using antibodies directed against CgA (eight evaluable cases). It is noteworthy that, *β*III-tubulin expression was also examined in one case of prostatic small cell carcinoma, an uncommon subtype of PCa with predominance of the NE phenotype (Yao *et al*, 2006). In this case, 100% of cancer cells expressed high levels of *β*III-tubulin (Figure 1F).

**Figure 2:**
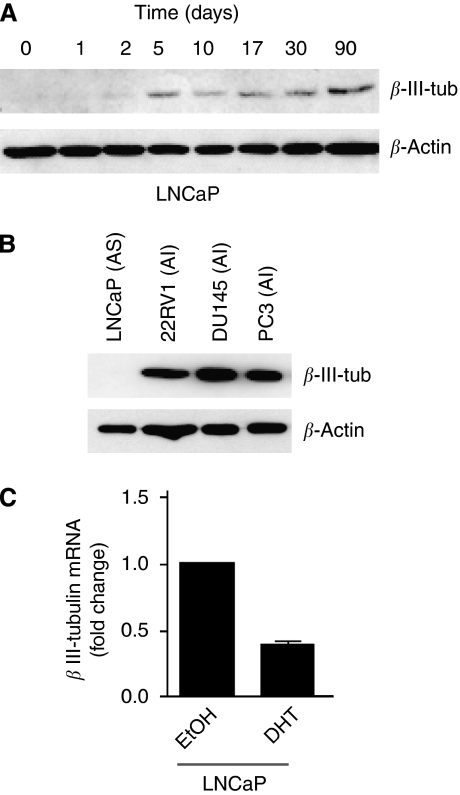
*β*III-tubulin expression is regulated by androgen depletion in LNCaP cultures and is expressed in androgen-independent PCa lines. (**A**) Time-course expression of *β*III-tubulin in LNCaPs cultivated in androgen-reduced medium. At day 0, monolayer cultured LNCaP cells were grown in the androgen-reduced medium. Each protein sample (30 *μ*g) was resolved to SDS–PAGE, transferred onto a polyvinylidene difluoride membrane (Millipore) and incubated with a monoclonal antibody against *β*III-tubulin or *β*-actin as an internal control. (**B**) The same procedure was applied to examine *β*III-tubulin expression in various PCa cell lines (LNCaP, 22Rv1, DU145 and PC3) maintained in RPMI 1640 supplemented with 10% fetal bovine serum (FBS), AS, androgen-sensitive; AI, androgen-independent. (**C**) *β*III-tubulin mRNA is reduced on 10 days of stimulation by 10 nM DHT in LNCaP cells relative to the expression in cells maintained in the androgen-reduced medium. Columns, mean±s.e.m.

A trans-differentiation phenomenon is observed in cultured androgen-sensitive PCa cells when they are maintained in a medium depleted of androgens (Burchardt *et al*, 1999), and therefore we examined, using western blot analysis, whether androgen-sensitive human prostate cancer LNCaP cells upregulate *β*III-tubulin when they are switched to androgen-deficient medium. Figure 2A shows that the steady-state levels of *β*III-tubulin protein were strikingly elevated in the days following growth in the androgen-depleted medium with persistent expression detected up to 3 months under these conditions. The ability of growth in the androgen-depleted medium to upregulate *β*III-tubulin protein expression was independent of the passage number of the LNCaP cells, as we obtained similar results using either low- (P25) or high-passage (P80) cells (data not shown). In addition, *β*III-tubulin protein was expressed in various androgen-independent human PCa cell lines, including 22Rv1, PC3 and DU145 after western blot analysis of protein extracts from these cells (Figure 2B). These data agree with a previous finding that *β*III-tubulin protein expression could be upregulated in LNCaP cells cultured in the androgen-deficient medium for 4 days or after transient suppression of androgen receptor expression using siRNA (Wright *et al*, 2003). We then tested the effect of the AR agonist, DHT on *β*III-tubulin mRNA expression. To this end, LNCaP cells were incubated for 10 days in an androgen-depleted medium supplemented with 10 nM DHT or with vehicle (ethanol, EtOH). The expression of *β*III-tubulin was partially abrogated in chronic DHT-treated cells (Figure 2C).

**Figure 3:**
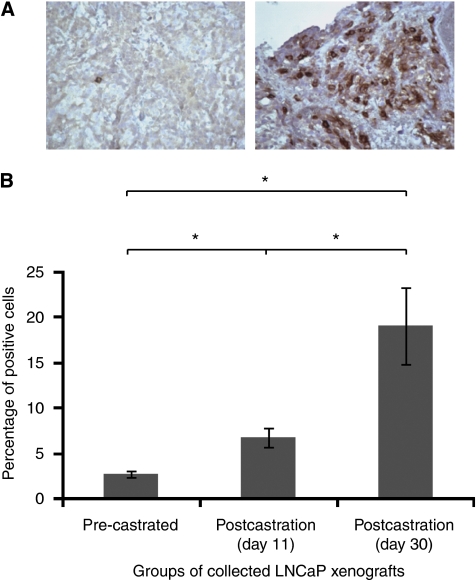
*β*III-tubulin expression increases after androgen depletion in LNCaP xenografts. (**A**) Representative immunostains in LNCaP xenografts with antibody to *β*III-tubulin. Immunohistochemical stains show weak or absent staining for LNCaP tumours from a non-castrated mouse (left), while a high proportion of positive cells with intense staining is detected 30 days after castration (right); original magnification, × 200. (**B**) Means of positive cells for *β*III-tubulin in each group. Kruskal–Wallis test (*P*=0.002) and Mann–Whitney test were used for statistical analysis of the immunohistochemistry scoring results. Columns mean; bars, s.e. ^*^P<0.05.

Finally, we sought to extend this observation by analysing *β*III-tubulin expression in LNCaP tumours after xenografting into athymic male nude mice. Five mice bearing tumours between 200 and 300 mm^3^ were killed and the tumour specimens were collected, fixed and embedded for sectioning. In all, 10 other mice with tumours of the same size were surgically castrated and then killed at day 11 or 30 post castration to retrieve and process the tumours for immunostaining. Both the pre-castrated and castrated levels of *β*III-tubulin were immunohistochemically analysed in various LNCaP tumours using the same parameters that were used for human tissues. Our analysis showed that the percentage of *β*III-tubulin-positive staining cells was significantly elevated in tumour cells from castrated groups in a time-dependent manner. Although only 2.7% of the tumour cells were found to be *β*III-tubulin positive in the tumour specimens obtained from the pre-castrated group, 6.8 or 19.1% of tumour cells were considered to be positive for *β*III-tubulin at 11 or 30 days after castration, respectively (Figure 3A and B). In summary, this study of cultured and engrafted human prostate cancer cells supports the idea that hormone therapy leading to the development of CRPC increases the expression of *β*III-tubulin in cancer cells.

## Discussion

This study for the first time provided definitive evidence that *β*III-tubulin is differentially expressed at the different stages of PCa progression. One notable aspect of our observations with regard to a relationship between *β*III-tubulin expression and hormone therapy for PCa is the possibility that *β*III-tubulin expression contributes to the aggressiveness of hormone-treated or castration-resistant PCa similar to the reports for other solid tumours. However, studies investigating the functional ablation of *β*III-tubulin functions in this setting are required to demonstrate conclusively its functional requirement for the emergence of castration-resistant tumours. Additional studies are also required to better define the mechanisms monitoring *β*III-tubulin expression. Advanced PCa and castration-resistant PCa have been associated with an attenuated androgen signalling signature (Tomlins *et al*, 2007). This information further supports androgen regulation and the increased expression of *β*III-tubulin in castration-resistant tumours. Interestingly, one recent study provided evidence for a potential role of oestradiol and oestrogen receptor (ER) in regulating *β*III-tubulin in breast cancer cells (Saussede-Aim *et al*, 2009). Although it remains to be determined as to whether this regulation implies a genomic or a non-genomic effect of ER, this observation could be of interest in PCa as oestrogen-dependent signature has been linked to aggressive characteristics in this tissue (Bonkhoff *et al*, 2001; Setlur *et al*, 2008). Future work will explore these questions. Recent data also indicate that hypoxia may stimulate *β*III-tubulin expression in the A2780 ovarian cancer cells (Raspaglio *et al*, 2008). We previously observed that hypoxia response can be altered in CRPC tumours and cell lines (Anastasiadis *et al*, 2002). This may highlight an alternative mechanism through which *β*III-tubulin expression is increased in those tumours.

In the hormone-naive PCa group, *β*III-tubulin expression in cancer cells was found to be restricted to a small number of prostate cancer samples. Our results contrast with a previous report showing that *β*III-tubulin was consistently detectable in HNPC, although at a relatively low level of expression that was comparable with normal prostate epithelial cells (Ranganathan *et al*, 1997). This difference might be attributable to a more stringent immunostaining protocol. Future studies including larger patient cohorts would help to determine whether *β*III-tubulin-positive tumours could represent a particular subtype of PCa. In addition, it will be of particular interest to examine the potential value of *β*III-tubulin in predicting response to microtubule-targeting agents in patients with prostate cancer. This knowledge may be useful for a better management of patients in that it could help to refine decision criteria and to better select patients who are more likely to benefit from chemotherapy regimens. We also believe that one appealing aspect of the association between *β*III-tubulin expression and hormone therapy for PCa is the indication that anti-cancer chemotherapeutic drugs that preferentially target microtubules with a *β*III-tubulin component might have greater value for the treatment of CRPC than would docetaxel, which is currently recommended. Whereas we already know that docetaxel therapy does provide some benefit for CRPC patients, this benefit is generally limited to a few months of additional survival (Petrylak *et al*, 2004; Tannock *et al*, 2004). Clearly, the evidence that *β*III-tubulin can modulate the sensitivity of cancer cells to standard taxane-based cancer therapy gives reason to consider whether other microtubule-targeting drugs with greater efficacy against cancer cells that express *β*III-tubulin might be more effective in this regard. One candidate for this type of approach may be the epothilone B analogue, ixabepilone, which is being tested against various solid tumours with *β*III-tubulin expression (Rivera *et al*, 2008; Dumontet *et al*, 2009) and that has already demonstrated activity in CRPCs, although it was with no consideration of *β*III-tubulin status (Galsky *et al*, 2005). Our results give strong reason to consider *β*III-tubulin status and to further test this therapeutic for CRPC patients.

## Figures and Tables

**Table 1:** *β*III-tubulin expression before and after hormone therapy

	***β*III-tubulin negative**	***β*III-tubulin positive**
**Prostate carcinoma**	**No. of samples (%)**	**No. of samples (%)**
Hormone-naive PCa (HNPC)	71 (95.9)	3 (4.1)
Hormone-therapy-treated PCa (HTPC)	18 (75)	6 (25)
Castration-resistant PCa (CRPC)	16 (40)	24 (60)
		
*Pearson's χ*^*2*^ *test*:	*P*<0.00001	
		
*Fisher's exact test*:		
HNPC/HTPC	*P*=0.0186	
HTPC/CRPC	*P*=0.0285	
HNPC/CRPC	*P*<0.000001	

Abbreviation: PCa=prostate carcinoma.

*χ*^*2*^ and Fisher's exact tests were used to assess associations between hormone and *β*III-tubulin status. For *post hoc* comparisons, the Bonferroni correction was applied. All statistical tests used a two-tailed *α*=0.05 level of significance.
