# Supplementary material for: Increased expression of class III β-tubulin in castration-resistant human prostate cancer
Source: Br J Cancer. 2009 Aug 18;101(6):951–6. doi: 10.1038/sj.bjc.6605245 (PMC2743364; doi:10.1038/sj.bjc.6605245)
Supplement: Supplementary Table 1 [file 6605245x1.doc]

| **Supplementary Table S1**: βIII-Tubulin expression before and after hormone therapy | | | | |
| --- | --- | --- | --- | --- |
|  | **Intensity of**  **immunostaining** | **No. of samples of** : | | |
|  |  | **Hormone Naïve**  **PCa** | **Hormone Therapy treated (3 months)**  **PCa** | **Castration Resistant**  **PCa** |
| **βIII-tubulin negative** | 0 | 67 | 9 | 3 |
|  | 1 | 2 | 3 | 7 |
|  | 2* | 2 | 4 | 2 |
|  | 3* | 0 | 2 | 4 |
| **βIII-tubulin positive** | 2 | 2 | 3 | 5 |
|  | 3 | 1 | 3 | 19 |
| **Total no. of samples** |  | 74 | 24 | 40 |

* The staining intensity was 2 or 3 in less than 10% neoplastic cells
